# Interprofessional KOPAL case conferences on patients with non-oncological palliative care needs – a qualitative analysis of interaction

**DOI:** 10.1186/s12904-026-02024-0

**Published:** 2026-02-26

**Authors:** Jan Philip Weber, Christiane Müller, Nadine Janis Pohontsch, Silke Böttcher, Uta Sekanina, Franziska Schade, Eva Hummers, Martin Scherer, Gabriella Marx, Stephanie Stiel

**Affiliations:** 1 Institute for General Practice and Palliative Care, Hannover Medical School, Carl- Neuberg-Straße 1, Hannover, 30625 Germany; 2 Department of General Practice, University Medical Center Goettingen, Robert- Koch-Straße 40, Goettingen, 37075 Germany; 3 Department of General Practice and Primary Care, University Medical Center Hamburg-Eppendorf, Martinistraße 52, Hamburg, 20251 Germany; 4 Department of Health Services Research, Division General Practice / Family Medicine, Carl von Ossietzky University of Oldenburg, Faculty VI Medicine and Health Sciences, Ammerländer Heerstrasse 114-118, Oldenburg, 26129 Germany; 5 Department of Palliative Medicine, University Medical Center Goettingen, Robert- Koch-Straße 40, Goettingen, 37075 Germany

**Keywords:** End of life, Ambulatory, Primary care, Home care services, Case conference, Teamwork

## Abstract

**Background:**

In Germany, patients with chronic non-malignant diseases (CNMD) are predominantly treated by general practitioners (GPs). Yet, little is known about communication between GPs and specialist palliative home care (SPHC) teams in terms of care needs and care planning for patients. Our analysis therefore aims at GPs´, SPHC nurses’ and SPHC physician’s interaction during case conferences (CCs) on patients with CNMD following an initial comprehensive palliative care consultation.

**Methods:**

A total of 78 CCs were conducted between January 2020 and March 2021 by telephone and recorded by non-participating observers using pre-categorized protocol forms. Content, course and patterns of communication of the participating 25 GPs, 16 SPHC nurses and 11 SPHC physicians are investigated by secondary document analysis with methodical reference to Kuckartz’s structuring content analysis.

**Results:**

CCs were characterized by a cooperative and welcoming atmosphere, focussing on the patient’s situation. CCs were mainly started and facilitated by the SPHC nurses. Most GPs showed great interest in nurses’ narrations and suggestions for improvement. GPs received important new information about their patients in some CCs. SPHC physicians mainly addressed medication regimens and emergency prevention.

**Conclusions:**

CCs’ participants interacted constructively in a positive atmosphere on an equal level. SPHC nurses hold a central role. We conclude that interprofessional CCs might support the improvement of health care of chronically ill patients by common reflection and the development of therapy goals and care plans. Increased structuring of CCs might trigger more specific treatment plans.

**Trial registration:**

KOPAL is registered on the German clinical trial register “Deutsches Register Klinischer Studien (DRKS)” (registration number DRKS00017795 17 November 2021, V.05).

## Background

The vast majority of patients with palliative care needs are cared for in their homes by their general practitioner (GP) and a general nursing home care service team [1]. About a quarter of those patients are in need of specialist palliative home care (SPHC) [2, 3]. These SPHC teams, consisting of specialized nurses and physicians, therefore provide additional medical and nursing care to patients with complex needs, trying to avoid hospital admissions [4]. According to statistics of one German statutory health insurance, 13% of around 96.000 deceased adult members received SPHC support during the last 6 months of their life in 2016 [5].

In the German context specialized nurses in the field of palliative care have 160 hours training in addition to their standard three years vocational training. Unlike in many other countries, to date most German nurses are not trained academically. Their courses consist of a mixture of theoretical and practical training organized and administered by public professional schools and work sites. Nurse practitioners or nurses performing tasks in substitution of physicians do not exist. However, draft legislation on the substitution of medical services is currently being discussed. In general, SPHC physicians are specialized in internal care or general practice and pass additional exams on palliative care after completing a 160 hours of training course.

Interprofessional interaction between nurses and physicians is generally perceived as challenging in Germany [6]. Although the evidence underpinning a strained relation is very little and mainly refers to hospital settings. Especially nurses think their work is not appreciated by physicians, for instance because appointments aren’t kept [7] or physicians’ communication is perceived as disrespectful [8].

Although oncological patients are considered to a greater extent compared to patients with chronic non-malignant diseases (CNMD) [9], chronic obstructive pulmonary disease (COPD), dementia and congestive heart failure are among the palliative conditions seen by German GPs [10].

Case conferences (CCs) of participants with different professional background are one tool for interprofessional collaboration in palliative care [11]. Patients suffering from the above-mentioned diseases are thought to benefit from their implementation as an interprofessional intervention in GPs primary palliative care provision [12], as available evidence is suggesting it [13–15]. Different models exist, mostly focusing on ethical or end of life care issues [e.g.^16^–^18^]. Most of those CCs include patient and her/his relatives [15, 19–21]. However, for the KOPAL study patients and relatives were excluded as single CCs of SPHC professionals and GPs had led to improvements for patients receiving palliative care before [19, 22]. German GPs and nurses considered CCs as a valuable tool to communicate about patients in primary care [13]. In an Australian pilot study, professionals assessed CCs positively; case management nurses reported an increase in knowledge and skills as well as a change in their care routines. Moreover, they perceived an improvement of GPs’ communication behaviours. Most of the GPs benefited from CCs as an improved time efficiency resulted [14]. On the other hand, GPs also have several reservations to CCs, such as hesitations to expand their routine face-to-face consultations with patients to other professionals. They also expressed doubts on whether CCs possible outcomes will justify the effort of organizing and fitting them into their already tight schedules [23]. However, in most investigations on CCs the results [14, 19, 22], content [24] or perception [20], but not the process were of interest. Qualitative research on the process of CCs is very rare.

### Research aim and questions

In the multicentre cluster-randomized controlled trial “Effectiveness of a specialist palliative home care nurse-patient consultation followed by an interprofessional telephone case conference compared with usual care among patients with non-oncological palliative care needs” (KOPAL) [12] interprofessional CCs were part of the intervention. KOPAL aims at reducing the number of hospitalizations of patients in the intervention group compared to control group (care as usual) patients as well as improve symptom control and increase quality of life [12]. The KOPAL intervention contains three components: following a (1) comprehensive SPHC nursing consultation at patients’ homes (or, due to SARS CoV2 pandemic, on the phone) and the (2) SPHC nurse’s presentation of findings to a SPHC physician, the (3) CCs between these two SPHC-team members and the patient’s GP take place.

The present sub-study investigates the interprofessional interaction between GP, SPHC nurse and SPHC physician (3) observed in CCs. Our understanding of interprofessional collaboration is based on the definition of the WHO: “Collaborative practice happens when multiple health workers from different professional backgrounds work together with patients, families, carers and communities to deliver the highest quality of care across settings” [25]. We chose this definition, despite CCs were performed by professionals only, as patients and their carers were involved directly in the precedent interaction (visit of SPHC nurse) and their perspectives were integrated into the CCs.

We intended to explore how participants organise themselves within the CCs: who leads the conversation? Which hierarchies can be observed? Who interacts with whom? What topics are addressed and by whom? Which intentions regarding the implementation of strategies are communicated at the end?

Therefore, this sub project aims to extend the understanding of interprofessional interactions, processes and proceedings between participants of KOPAL CCs in primary care. The investigation evaluates the course of conversation, analysis how CCs’ participants presented themselves (professional performance), describes the characteristics of interprofessional interactions and names CCs’ results.

## Methods

### Design

In this qualitative observation study, we explore the interprofessional communication of participants of KOPAL CCs as documented by the completed protocols of non-participating observations. Content analysis [26] was chosen as this method allows the structuring and characterisation of the process of CCs.

### Study material

The study material consists of three documents: The (1) KOPAL study protocol defines study background, aims, questions and methods of the KOPAL main study. It was basis for the study proposal, ethical approval and trial registration and is published elsewhere [12]. The (2) “KOPAL Conversation Guide” for the SPHC nurses’ conversation with the patients was developed based on the British ‘PEPSI COLA aide memoire’. The PEPSI COLA aide memoire is a holistic common assessment of supportive and palliative care needs of adults with cancer [27]. The (3) ‘pre-categorized KOPAL observation protocol form’ was developed and designed by the study-team and meant to support documentation of the content and course of conversation of the CCs. The categories are based on the expected topics and proceedings of case-conferences in the context of palliative outpatient care.

### Research setting

CCs were conducted between June 2020 and March 2021 via telephone within a two-week time slot after the respective SPHC nurses had conducted an individual conversation with the participants of the intervention group on the basis of the “KOPAL Conversation Guide” [12]. As patients were not eligible for SPHC yet, SPHC physician’s involvement in the individual patient conversation was not considered suitable. The CCs were technically organized by researchers of the respective study centers. Researchers were provided with a copy of the completed KOPAL Conversation Guide form and SPHC nurses were provided with the instruction to brief the SPHC physician prior to the CC, though it cannot be guaranteed that it took place in all cases. The chosen CC procedure is in accordance with § 3 section II of the German Social Security Code, Public Health Insurance [SGB V] where patients’ or relatives’ participation in the interprofessional collaboration for the initiation of SPHC care is not intended. Here CCs aimed at improving patient’s care by providing orientation to GPs and helped overcoming possible shortcomings in patient’s care. In contrast, in our study the GP was informed about the content of the palliative care consultation during the CC. A timeframe of 30 min for each patient was scheduled. The three participants discussed the patient’s health care situation without any further specification on sequence, topics or aims given from researcher’s side. Researchers neither facilitated the CCs nor were intended to respond to comments or questions.

### Participants and sampling

KOPALs CCs were limited to GPs, the SPHC nurse and the SPHC physician who were recruited for the KOPAL main study according to the inclusion and exclusion criteria [12].

### Data collection

A total of 78 CCs were observed and documented in written form by researchers of the study sites: three male (JW, MZ, TS) and four female researchers (NP, GM, US, SB) of different professional backgrounds: psychologist (NP), public health scientist (JW, SB), nursing scientist (MZ), master of arts in education for health professions (US), physician and sports scientist (TS), and sociologist (GM). While researchers did not contribute to the CC, participants were informed about researchers’ passive presence in the conference call. All researchers had experience in qualitative research (range 4 to 15 years) or received intensive training on data collection. After five initial observations the observation protocol was discussed regarding feasibility resulting in no need for any modifications. Prior to the study, no relationship was established between researchers and participants. However, participants knew about researcher’s status as employees of the respective study site and, in some cases, were aware of their professional background.

During the CCs the researchers took notes using the pre-categorized KOPAL protocol form. The form was meant to formalize observations and to secure intersubjective reproducibility and comprehensibility. The inherent guide for all observations was the following set of questions: What is the observation about? Who takes an active part? When, how often and to what extent does the observed phenomenon occur? What is the possible aim? Eight subject areas entailed questions and suggestions concerning the recording of the course and content of the CC discussion (Table 1). The categories context and methodological and theoretical reflections are not considered in the present analyses, as only a handful entries were found in the protocol. As a matter of quality control, three initial CCs were recorded by two observers and notes were compared and merged later.

**Table 1:** Categories and suggestions of observers’ case conference log-form

Observation
Professional Interaction • What is the actual course of interaction within the case conference (introduction, execution, problem solving, achieving consensus, facilitation, contributions)? • Who does what, how, with whom, when and where? • What happens in which course? • Are there incidences / situations / conflicts arising and if, how are they solved? • What type of contact can be observed? (atmosphere)
Role Behavior • Participants hierarchy • Distribution of roles • Who facilitates the discussion throughout the course of conversation? • Who advocates for the patient? • Who considers patients ’ needs? • Is any participant holding back? • Contribution time • Which constellations are to be observed?Topics • Which patient related topics are highlighted? • What is the subject of discussion? (remarkable quotes, transcript sequences of interaction) • What is not subject to discussion? • Has an important topic not been considered? • Have all fields ’ manual major topics been addressed? • Which different point of views are presented by whom? • Are other topics addressed (e.g. small talk at onset, other patients)?
Decision making • Are there different positions in terms of patients’ goalsetting for treatment and therapy? • Are decisions made commonly? • Is there an elaboration of alternative solutions? • Are all participants actively engaged in decision making? • Which significance is admitted to nursing perspective?
Results of Case Conference (further steps, treatment plan, what might be implemented, who will be involved? )
Please estimate to which extend the KOPAL Conversation Guide has been considered (deviation, omission etc.)
Context (what was the overall framing of the case conference, interruptions, unexpected occurrences)
Methodological reflection • Do observations relate to any methodological consequences? • Does my presence affect participants’ interaction (call for facilitation of discussion, interaction between participants and observer)? • What’s my position in the field?
Theoretical reflections on the participants’ interprofessional collaboration • How can observations preliminarily be reported? • Which interrelations can be assumed?

The hard copies of the completed observation protocols were stored in locked closets of the respective research sites. Scanned copies with pseudonyms only were stored as password protected 7-ZIP container in order to gain qualitative data analyses software accessibility.

### Data analysis

Data analysis was guided by Kuckartz structured content analysis [26] and conducted using MAXQDA 12 coding software. Following the review of fifteen observer protocols, a first coding system was drafted. Anchor samples were defined to guide the coding process.

Main categories were constituted deductively from CC protocol form categories (Table 1) and inductively drawn from handwritten observers’ notes. In accordance with Kuckartz’ [26] methodological guidelines with regard to the initial draft of the coding system, few main categories and subcodes were added throughout the coding process. All protocols were coded by two researchers (JW, CM) either simultaneously or in turns. Content and coding memos were written by both researchers accordingly. Codes and memos were discussed until agreement was achieved. Moreover, findings were reflected in the scope of a cross-consortia team meeting (JW, CM, GM, NP, FS) twice a month.

## Results

### Socio-demographic key data and case conference characteristics

The 78 observed CCs lasted on average 18 min (range 5 to 60 min) per patient. At least 33 of the patients discussed were female, 45 male. Sometimes several patients were discussed in one appointment. Due to an unexpected shift of appointment by CC participants five additional CCs were not observed and therefore not considered here. On average GPs participated in 3 CCs (range 1 to 7). Overall 36 patients being subject to CCs were diagnosed with congestive heart failure, 29 COPD and 21 dementia, while specification of multiple diagnoses was allowed. Socio-demographic key data of CC participants are summarised in Table 2.

**Table 2:** Socio-demographic key data of CC participants

General practitioners in CCs (*n* = 25)	
Age at baseline in years* (mean [min., max.])	53 [38, 64]
Sex, female (N [%])	14 [56]
Mean professional experience (years** [min., max.])	15 [2, 27]
Palliative care training prior to study (N [%])	5 [20]
SPHC nurses in CCs (*n* = 16)	
Age at baseline in years*** (mean [min., max.])	49 [31, 57]
Sex, female (N[%])	16 [100]
3 year-vocational nurse training (N [%])	13 [81.3]
3 year-vocational training in elderly care (N [%])	3 [18.8]
Additional academic training in health sciences (N [%])	3 [18.8]
SPHC physicians in CCs (*n* = 11)	
Age at baseline in years (mean [min., max.])	58 [42, 72]
Sex, female (N [%])	3 [27]
Qualification: General practice (*n* = 5), anesthesia (*n* = 3), internal medicine (*n* = 4), social medicine (*n* = 1) (specification of multiple qualifications allowed)	

*n = 24 (one value missing); ** *n* = 23 (two values missing); *** *n* = 15 (one value missing)

The majority of CCs were composed of a female GP, a female SPHC nurse and a male SPHC physician (*n* = 23) or a female SPHC physician (*n* = 19) respectively. In 22 CCs the combination of a single male GP, female SPHC nurse and male SPHC physician was almost as frequent (Table 3). In single CCs two GPs of different sex participated.

**Table 3:** Gender composition of CCs

Gender composition			
GP	SPHC nurse	SPHC physician	No. of CCs
female	female	female	19
female & male	female	female	4
female	female	male	23
male	female	female	7
male	male	female	2
male & female	female	female	1
male	female	male	22
No completed protocol form available			5
Total			83

### Technical and schedule related issues

In 12 CCs technical problems occurred in terms of a delayed start or acoustics. All issues were solved quickly. Medical participants arrived late (GPs and SPHC physicians) or had to leave ahead of time (only SHPC physicians) in 12 CCs. One time a SPHC nurse arrived late.

### Content related findings of the case conference protocol notes

The coding analysis revealed the six major areas of interaction “Onset of case conference”, “Professional performance”, “Course of case conference”, “Interprofessional interaction”, “Atmosphere of case conference” and “Results of case conferences” to be central for the description and understanding of interactions within CCs.

#### Onset of case conferences

Almost half of the completed observer protocols contained references on who had taken initiative at the onset of a CC. Conversation was most often started by the SPHC nurses (in 20 CCs), sometimes by the GP (in 6 CCs), and in rare cases by the SPHC physician (in 4 CCs). In the following, the SPHC nurse often reported course and findings of the initial patient’s consultation. Alternatively, the GP summarized the medical history of the patient and the nurses supplemented their observations.

#### Professional performance

GPs were well informed about the medical history of their patients, their social environment and the assistance patients received in their homes, as highlighted by the protocol notes. GPs were heavily involved in the provision of care and presented themselves as specialists on the medical and social matters of their patients. They were eager to defend their sovereignty with regard to the interpretation of those topics. According to observer notes, GPs related apparent knowledge gaps to organisational deficits in their office, e.g. the GP participating in the CC was not the GP primarily seeing the patient. Moreover, GPs were impressed by the additional information provided by the SPHC nurses. GPs benefited from the information and expressed their gratitude.

According to the notes, SPHC nurses strongly represented the nursing position within CCs as they particularly introduced patients’ care needs, analysed respective barriers, and suggested approaches to overcome them. They therefore often became an agent of information on the patients; for instance, when talking about prescriptions of medication or medical examinations by other specialists. Occasionally observers noted that SPHC nurses behaved rather “self-confidently” and characterized their communication style as dominant and not focused on nursing issues. Single GPs reacted cautiously to SPHC teams’ suggestions.

The third profession in the CCs, the SPHC physicians contributed to the discussion when they perceived the topic to be appropriate. They mainly addressed medication regimens and emergency prevention issues. In rare CCs nursing colleagues held back when SPHC physicians spoke and nursing topics were at risk of underrepresentation.

Many CC notes pointed out that all participants thought along patients’ perspectives. Yet SPHC nurses and GPs were more dedicated when arguing either commonly or separately for the patients’ perspective. In addition, relatives’ advocacy was mostly attributed to the SPHC nurses considering the thread of relatives’ physical and psychological exhaustion due to the labour-intensive patients’ care. In consequence, SPHC nurses made suggestions for relatives’ relief (e.g. additional support, psychological care, psychosocial support acquired by participation in self-help groups, clarification on patient’s medication, information on available counselling and outpatient care providers).

#### Course of case conferences

CCs were mostly facilitated by SPHC nurses. Apparently, they led larger sequences of the conversations; GPs directed CCs or SPHC physicians structured conversations only at times. One single protocol indicated no facilitation at all and participants interrupting each other constantly.

Considering completed protocols, allocation of contribution time varied considerably between CCs. In most CCs, SPHC nurses and GPs spoke most often. In a few CCs GPs remained in the background or had rather little contribution time. Sometimes SPHC nurses’ communication was characterized as a monologue. Sporadic notes indicated that the allocation of participants’ contribution time changed over the course of conversation, e.g. SPHC nurses communicated more at onset while SPHC physicians spoke more towards the end.

Other protocols pointed out that SPHC physicians acted rather restrained and had little contribution time. Sometimes they gently followed and joined the conversation and in some CCs they behaved completely passive. Some observers assumed SPHC physicians’ hesitation was attributed to the fact that they did not personally know the patient, but had been informed about the prior patient assessment by the SPHC nurses. However, in very few CCs SPHC physicians had the most contribution time.

Participants’ sex as an influencing factor on the allocation of contribution time was mentioned only rarely. Very few comments described male physicians to communicate rather dominant and female participants to behave more reserved.

Although SPHC nurses’ initial palliative care consultations were based on the topics of the KOPAL Conversation Guide in most CCs a reflection of the order of topics was not observed. Yet, sometimes the course of conversation considered parts of it.

#### Interprofessional interaction

According to the completed protocols the interprofessional communication mostly focused on the factual level and was characterized as an exchange in which all participants had the opportunity to contribute. Generally, participants were considerate of each other. Observers perceived the communication style to be constructive. Participants intensively communicated their patient-related observations and thoughts, and listened to each other´s contributions. Particularly, SPHC nurses and GPs appreciated each other’s contributions. They made precise suggestions for future practice and GPs often endorsed SPHC nurses´ proposals. In many CCs, all participants commonly searched for solutions to improve patients’ care.

With regard to the communication between the physicians, SPHC physicians directly responded upon GPs specific requests in some CCs. Very seldom disagreements concerning preferred medication approaches were recorded.

For the most part, communication patterns were not shaped by hierarchy or a constantly dominating behaviour of a single profession. About a quarter of protocols indicated that SPHC physicians and GPs communicated solely with each other for some time, while the SPHC nurse did not speak. In rare cases SPHC members hardly appeared as a team during CCs.

According to notes the SPHC teams hardly expressed outright criticism concerning GPs´ performance. They implicitly questioned GPs´ position as the leading care provider only in single CCs. Even in apparent gaps in the prior provision of medical care, the SPHC team members reacted constructively; seeking to solve problems without blaming the GP. Rarely GPs defended themselves or justified identified shortcomings. Sometimes GPs even asked for feedback concerning their prior therapy. More notes described GPs using the opportunity to clarify general question on SPHC.

#### Atmosphere of case conferences

The atmosphere of CCs was characterized to be predominantly very “friendly” and “appreciative”. Moreover, it was often mentioned that no “critical situations” or “conflicts” occurred. At times the conversation was even used for small talk or the discussion of patients not being the subject of the current CC.

In contrast, notes indicating unpleasant interaction were very rare and limited to short sequences in which participants were cutting one another off. Even more rarely CC conversations were “hectic” or “exhausting”, “speedy” or under “time pressure”, the ladder especially when several patients were discussed at one appointment.

#### Results of case conferences

During the CCs, particularly SPHC nurses and GPs made precise suggestions for future care. In many CCs, all participants were commonly searching for solutions for the improvement of patients’ care.

According to the field notes, GPs considered further procedures for patients mostly related to medical care: advance care planning, the implementation of emergency plans, the adjustment of current medication or the referral to a specialist. Other areas of future activity were the implementation of nursing home care services as well as psychosocial and non-medical therapeutic interventions. Future clinical diagnostics were less often mentioned (Table 4). In some CCs, the discussion solely focused on SPHC nurses’ findings without a deeper discussion or an assignment of responsibilities. In other CCs no active search for (alternative) solutions was recorded.

**Table 4:**
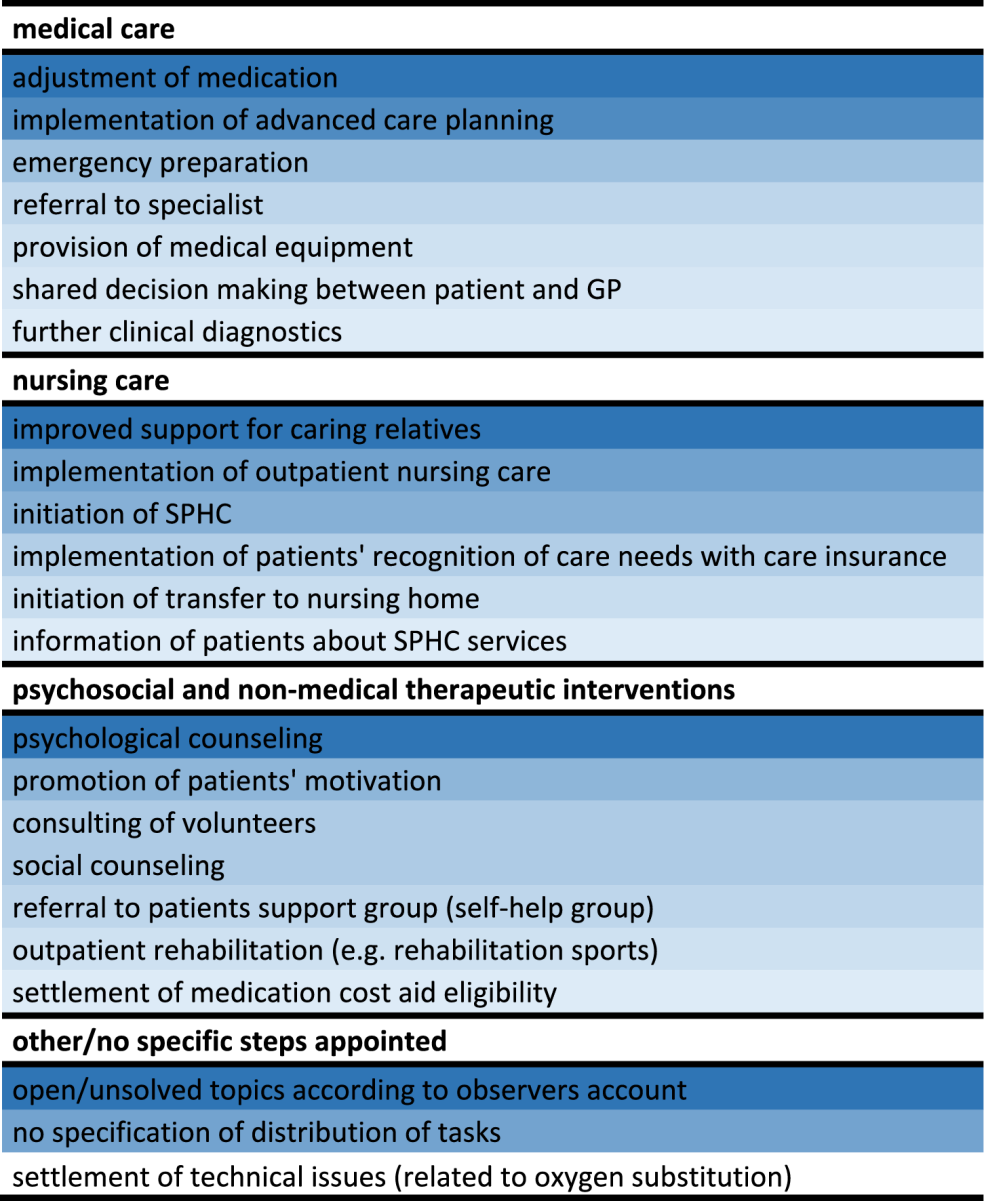
Heatmap of observed case conference results: frequent (dark blue), less frequent (light blue)

## Discussion

### Original findings

Telephone CCs were perceived to be conducted in a positive and trustful atmosphere. CCs usually started with a report of the patient’s situation by the SPHC nurse who mainly led the CC. GPs were observed to be very interested in nurses’ suggestions for an improved health care. SPHC physicians contributed mainly to the topics of medication and emergency regimens. Finalisation of CCs varied: in some GPs stated their next steps, in others no further proceedings were communicated. The topics of the KOPAL Conversation Guide were considered varying intensity.

The KOPAL CCs directly started with patient’s issues. Remarkably, none of the protocol forms indicated any sort of negotiations on CCs’ agenda. This goes along with the widespread absence of CCs’ participants own introduction during the opening phase. Thus, the specifics of the intervention may have contributed to this as most participants joined more than one CC and were introduced to each other by the researcher at first. Some participants knew each other from previous collaborations with regard to oncological patients.

We could see that professional roles and the set of topics were not clarified at all – findings that had been reported by Shelby-James et al. [15] before. Their study group observed that participants of CCs did not negotiate roles with regard to facilitation or general tasks within CCs even though relatives and informal caregiver were joining their CCs.

Similarly, the distribution of contribution time was never negotiated or limited by CCs´ participants. Possibly, the extent of contributions varied according to the factual needs or participants’ ability to contribute. Whether the expense allowance of 60€/per CC had any impact on the individual contribution time or the overall length of the CCs should be considered and investigated as an influencing factor in future trials. Albeit the lack of clarification of a time frame or time for contributions, it cannot be expected, that contribution time reflects contribution content.

Additionally, we noticed the absence of notes on (extensive) hierarchical rejection of any profession or single participants. Instead, the conversations were perceived as mainly fact driven and held in a trustful, respectful, informative and constructive atmosphere that even allowed to address shortcomings in care almost without forcing GPs into defence. However, the absence of (open) conflicts is not a unique finding as it was reported before by a study from Japan that investigated interprofessional CCs on ethical dilemmas in end of life care [24]. The participating physicians, nurses and care workers were observed to have interacted without conflict, even though their professional positions regarding the cases discussed differed [24]. We presume that the outpatient setting and the overall limited cooperation and familiarity between GPs and SPHC members might play a role since there is evidence for the reproduction of hierarchical team conflict in ethical CCs elsewhere [28].

At first glance, findings entailed few surprises with regard to participant’s professional behaviour and performance. With reference to the traditionally rather weak position of the nursing profession in Germany [29], observations described a boost of confidence herein: according to the analysed observer notes most SPHC nurses behaved very confident during CCs and vice versa were respected as eye-to-eye partners by the physicians. In most CCs they acted as a facilitator and were involved over the entire course of the CC. Reasons might be found in nurses’ confidence of their own expertise, their SPHC specialization and the additional academic training some of them were holding. Although several courses of nursing studies exist in Germany, the professions’ academisation is still in process and vocational training continues to be the standard qualification [30]. Moreover, 14 of 16 SPHC nurses had a leading or coordinating position within their SPHC team. However, the study design was in favour of them: In KOPAL’s intervention SPHC nurses had a key position. They were free to structure their reports according to their own preferences and given the opportunity to act as a consultant on GP´s prior treatment. Considered that, lots of work needs to be done to improve nurse’s position and to reduce the hierarchical culture in medical care in Germany (1). KOPAL’s CCs supported nurses in taking responsible tasks and might help to catch up to international standards.

During the interprofessional exchange, GPs often tried to keep the sovereignty of the discussion of the respective patient and concurrently presented themselves open for consultancy. Herein the before cited study conducted by Shelby-James et al. differs as in their CCs leadership was more frequently taken by a physician, while the nurse acted more reluctant [15]. In some CCs, GPs received important new information on their patient by the other professionals in the CC. Herein findings of the present study are similar to those reported by Shelby-James et al. from an Australian investigation, in which GPs also received more recent information or requested further information in the CCs [15]. In our study GPs appreciated the provision of further information on their patients as it could be used for the improvement of patient’s care.

Whether SPHC physicians’ reluctance resulted from the dominance of care related topics instead of medical or medication related issues, or the fact that they did not personally know the patients might be revealed by the qualitative evaluation of the KOPAL study. Hence, it should be discussed to which extent SPHC physicians should be regularly considered in a future implementation of interprofessional CCs in regular primary palliative care. Our results indicate that their participation upon request and depending on the initial palliative care consultation findings might be reasonable.

Though gender aspects were hardly addressed by observer’s notes, their potential impact on the CCs cannot be ruled out. Various background and a gender mix were meant to support respective sensitiveness among researchers.

The rather positive picture of the interprofessional communication in CCs might only be half of the account, as the outcomes for patient’s further health care were ambiguous. Only a few notes indicated a joint development of precise intervention plans for the patient or the set of timestamps for their accomplishment. Shelby-James [15] reported similar results regarding CCs on end-of-life care, in which, after vivid discussion among participants, also no summary was drawn. A reason for the omissions in the KOPAL study might be, that the determination of an action plan was not claimed by the study design and in most cases patient’s health status did not require the implementation of SPHC yet. In consequence, participants were not in need to negotiate common proceedings or further SPHC steps in the CCs. In any case, participants missed the chance to agree on low threshold consultations or prescriptions and thus the establishment of future relationships.

### Implications for research and practice

In KOPALs’ CCs, SPHC nurses had a key position with regard to the presentation of patient’s situation, the suggestions on further treatment and the overall facilitation of the course of CCs. The accomplishment of these tasks is a reference for SPHC nurses’ competencies, especially since only three of them hold an academic degree in addition to their completed standard vocational nurse or elderly care training. It therefore appears reasonable to extend their scope of responsibility and to assign them to more tasks at the intersection of primary palliative care and SPHC.

With respect to KOPALs’ CC design, SPHC nurses’ commitment implicates that the designation of a moderator was actually not necessary. Based on our experience, we would recommend a modest modification with regard to the technical pre-structuring of the CCs in future CC implementation into regular healthcare. In a theoretical framework for the training of interprofessional CCs the (1) opening phase allows participants to clarify their roles and the (2) core phase encompasses the joint description and negotiation of relevant health related aspects. In the (3) termination phase participants tackle relational issues and agree on patient’s future healthcare plan [31]. Though we explicitly decided not to provide a fixed agenda for our CCs, more specifications on participant’s tasks or a general agenda might be useful to support CC participants in developing a defined treatment plan in future clinical practice. For instance, Halcomb et al. [20] highlight the importance of a framework as a tool to effectively conduct CCs. Their framework contains pre-defined and disclosed professional roles to avoid confusion in terms of participants’ expectations, especially when they have no history of cooperation and are uncertain about each other’s skills [20].

In addition, the provision of information about patient’s situation prior to CCs might further raise CCs’ effectiveness. This assumption is consistent with conclusions drawn elsewhere: “…all participants […should be] provided with a summary of recent clinical history (including tests, medications, involvement of other healthcare services and current or future needs)” [15], p. 609 f., alike: [32].

From a methodical point of view and despite the rapid advancement of qualitative health care research, methods on the structured analysis of observers’ notes are extremely rare [33, 34]. The current discussion rather focuses on technical improvements of conducting observational field notes, whereas their inherent selection, valuation and the assignment of their relevance are barely reflected [33, 35]. Further theoretical and empirical research is needed to investigate the particularities of field notes analysis.

### Study limitations

One limitation of our study might be found in a selection bias as participants of the study are expected to be keen of the addressed topics SPHC, CCs and interprofessional interactions. In addition, social desirability of behavior might have an impact on findings. Participants knew that they were observed and therefore possibly behaved more gently than they usually would. This might explain why hardly any tension was observed between GPs and SPHC team members, though field and scientific reports often suggest the opposite [36–38]. On the other hand, observers did not think that their presence was considered at all by participants in most CCs and SPHC team members knew that their task was limited to consultancy.

The apparent improvement in nurses’ position as an additional effect of CCs as well as other findings may be specific to the KOPAL protocol and the German healthcare context, but not necessarily applicable to other countries.

Other possible limitations of our study are related to the method of data collection. Different to other projects [e.g. 14, 24], KOPALs’ CCs were conducted on the phone instead of in face-to-face encounters. This might influence the intensity of the interactions negatively, if participants were unsettled or not experienced with it. On the other hand, palliative telephone CCs were successfully conducted [21, 22] and rated time effectively by GPs [39] before.

From a methodological point of view, the depth and the interpretive richness of the analyses conducted might have been limited by the fact that the qualitative observations rely on structured observer notes rather than verbatim transcripts.

## Conclusions

Interprofessional CCs for palliative outpatients based on a SPHC initial comprehensive palliative care consultation in patients’ homes are well feasible and generally, participants interacted constructively in a positive atmosphere on an equal level. SPHC nurses proved their competencies to act as facilitators. In general, they had a key role within CCs in our study and their position in the care of palliative patients should be strengthened. Moreover, the use of a rough agenda for the process of CCs might result in a more practical planning of further steps.

We conclude that interprofessional CCs might support the improvement of health care of chronically ill patients’ by common reflection and the development of therapy goals and care plans. Results of our study should contribute to a further improvement and a higher quality of palliative patient’s health care.

## Data Availability

The datasets analyzed in this study are available from the corresponding author upon reasonable request.
